# Hadron production models’ prediction for *p*_*T*_ distribution of charged hadrons in pp interactions at 7 *TeV*

**DOI:** 10.1038/s41598-019-48272-4

**Published:** 2019-08-14

**Authors:** S. Ullah, M. Ajaz, Z. Wazir, Y. Ali, K. H. Khan, H. Younis

**Affiliations:** 1 Department of Physics, Abdul Wali Khan University Mardan, Mardan, 23200 Pakistan; 2 Department of Physics, International Islamic University Islamabad, Islamabad, 44000 Pakistan; 3 Department of Physics, COMSATS University, Islamabad, 44000 Pakistan; 4 Department of Physics, Women University of Azad Jammu and Kashmir, Bagh, Pakistan

**Keywords:** Phenomenology, Theoretical particle physics

## Abstract

The yield of transverse momentum (*p*_*T*_) spectra of integrated hadrons and their ratios produced in *pp* collisions at 7 TeV are reported using DPMJET-III, EPOS1.99, EPOS-LHC, HIJING1.383, QGSJETII-04, and Sibyll2.3c models. The models’ predictions are compared with the ALICE measurements obtained at mid rapidity and in the *p*_*T*_ range from 0.3–6 GeV/*c*, 0.2–6 GeV/*c*, and 0.1–3 GeV/*c* for protons, kaons and pions respectively. Compared to the experimental data, the EPOS1.99 and EPOS-LHC models predict the yield of pion very well while reproducing yields of kaon and proton at low *p*_*T*_ only. The DPMJET-III model predicts the yield of pions, kaons and protons only at intermediate value of *p*_*T*_. The HIJING and Sibyll models describe the integrated yield of pion well at high *p*_*T*_ only. The QGSJETII model predicts the yield of kaon for almost the whole *p*_*T*_ range. EPOS1.99 and Sibyll models reproduce excellent prediction of the *K*/π ratio over the entire range of *p*_*T*_ whereas the EPOS-LHC, HIJING and DPMJET models underpredict with increasing order of discrepancy respectively. The EPOS1.99, EPOS-LHC, and Sibyll models predict the *p*/π ratio for low values of *p*_*T*,_ but overpredict at high values, while the DPMJET-III and HIJING models underpredict over the entire range of *p*_*T*_. Although the models’ predictions are mostly consistent with the ALICE measurements but none of the models completely describe the entire distribution for the integrated yield of hadron and their ratios.

## Introduction

The measurements of hadron production have an extended record in high energy nuclear and particle physics^1–13^. In order to test soft parton interactions and hadronization processes predicted by non-perturbative quantum chromodynamics, the transverse momentum (*p*_T_) spectra and absolute yields of hadrons are used by Monte Carlo (MC) event generators^14^. We get important information on multi-parton interactions and other final state effects from the dependence of the yield and *p*_T_ distribution on the hardness in *pp* collisions. The identified hadron distributions in *pp* collisions also provide an important reference to the findings of high-energy heavy-ion analysis, where the spectral shape and yield of hadron species are modified by the final state effect. The interactions responsible for *pp* collisions and subsequent hadronization process at the LHC can be understood using the quantum chromodynamics^15^. Phenomenological models must be employed, as precise calculations are difficult to perform in the non-perturbative regime. Monte Carlo event generators based on these models need to be tuned, to reproduce experimental observables.

The present analysis is focused on the study of *p*_*T*_ spectra of the integrated yield of π (π^+^ + π^−^), *K* (*K*^+^ + *K*^*−*^), proton (proton + anti-proton) production, and the *K*/π and *p*/π ratios in proton-proton (*pp*) interactions at 7 *TeV* using DPMJET-III, HIJING1.383, EPOS, QGSJETII-04, and Sibyll2.3c models, and their comparison with the data from the ALICE experiment^11^. The analysis, in future, will be extended to heavy ion collision for the study of strongly interacting matter and to search for a signal on the onset of deconfinement.

## The Method and Models

*p*_*T*_ distributions of differential yields of integrated hadrons (sum of positively and negatively charged hadron) and their ratios, normalized by the total number of inelastic events, simulated for *pp* interactions at 7 *TeV* in the *p*_*T*_ range from 0.1–3 GeV/*c* for pions, from 0.2–6 GeV/*c* for kaons, and from 0.3–6 GeV/*c* for protons with 0 < y < 0.5 are presented here. Model simulations of 1M events using DPMJET, HIJING1.383, EPOS1.99, EPOS-LHC, QGSJETII-04 and Sibyll2.3c are compared with the measurements of experimental data from the ALICE experiment^11^. Brief description of the models is given below while references given against each are referred for complete description of each model.

DPMJET-III^16^ is a Monte Carlo code constructed on the Dual Parton Model^17^ combing the features of DPMJET-II^18^ Dtunuc-2^19,20^, and Phojet1.12^21^. DPMJET-III is a multipurpose code that allows for the simulation of photon-photon, photon-hadron, photon-nucleus, hadron-hadron (*hh*), hadron-nucleus (*hA*), and nucleus-nucleus (*AA*) interactions from a few GeV up to the highest energies cosmic rays^16^. In our analysis, we used DPMJETIII.17-1, which is an updated version of DPMJET-2.55 where few parameters were changed to reproduce TOTEM cross section^22^. The model is the Tevatron version while the new LHC version is not public yet.

HIJING is a hadron production model based on Monte Carlo simulation that allows high energy *hh*, *hA*, and *AA* collisions^23^. The model is aimed particularly to study jets and related particle production based on QCD-inspired models. HIJING includes mechanisms such as soft excitation, production of multiple minijets, interactions of jets in dense hadronic matter, and nuclear shadowing of parton distribution functions^24^. In our simulation, we used HIJING 1.383 with default parameters. The model is using Pythia 5.3 to generate kinetic variables and Jetset 7.2 for jet fragmentation.

EPOS is a hadronic interaction Monte Carlo event generator used for minimum bias interactions of heavy ion as well as cosmic ray air shower simulations^25,26^. EPOS is also used for centrality dependent heavy-ion collision. Energy conservation is used by EPOS model at amplitude level. The EPOS1.99 model is tuned to data up to Tevatron energies. An updated version of EPOS for Large Hadron Collider (LHC) energies, is EPOS-LHC, where a different parametrization of flow has been introduced in *pp* collisions compared to heavy-ion producing large volume. EPOS-LHC model is tuned to LHC data up to 8 TeV but the 13 TeV data tune is still missing in it. The model, utilizing colour exchange mechanism of string excitation, is tuned to LHC data recorded in 2012. It is based on the Gribov Regge approach where the same flow parameterization is used as is used in EPOS1.99. EPOS-LHC can reproduce the minimum bias results for all those particles that are having transverse momentum, ranging from 0 to a few GeV/c^27,28^. Beside EPOS-LHC, there is another parallel development of the EPOS model named EPOS 3, based on the same approach as EPOS1.99, used for collectivity in heavy ion collision.

QGSJET (Quark Gluon String model with JETs) is a Monte Carlo generator widely used in many collaborations, and relies on the physics picture of the Quark-Gluon String model^29^. The Gribov’s Reggeon calculus approach is used in the QGSJET model for hadronic and nuclear collisions as multiple scattering processes^30,31^. Like EPOS-LHC, QGSJET is also tuned to several accelerator data including LHC data up to 8 TeV. No energy conservation in Sibyll and QGSJET is used for cross section calculations. Sibyll is a fast Monte Carlo event generator used for air shower simulations^32^. The model is able to describe features of hadronic particle production. It is a QCD inspired model that includes multiple interactions, hard as well a soft scattering, string fragmentation and saturation effects. Sibyll 2.1^33^, that was tuned to TEVATRAN data is updated to Sibyll 2.3c^34^, to describe the NA49 data. The new version further includes charm production, baryon pair production and beam remnants. The model is tuned to NA61 data and to LHC data at 8 TeV.

The analysis is performed using the RIVET Monte Carlo analysis tool^35^, which has a big code library to compare the results available on HEPDATA with the Monte Carlo event generators. It is based on the HepMC for the universal event record format^36^, on the library of LHAPDF for parton densities^37,38^, and on the Fastjet package for jet clustering^39,40^.

## Results and Discussion

**Figure 1:**
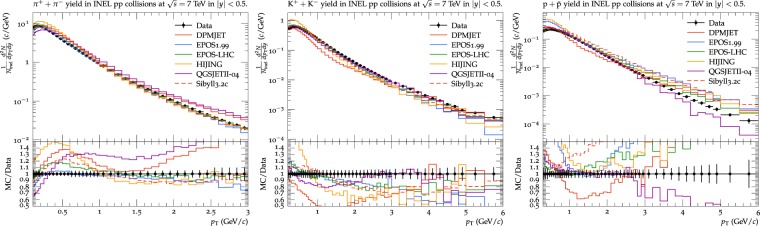
Transverse momentum spectra of the integrated yield of pion, kaons and protons by DPMJET-III, EPOS1.99, HIJING1.383, EPOS-LHC, QGSJETII-04 and Sibyll2.3c in pp collisions at 7 TeV are compared with the measurements of experimental data from the ALICE experiment. Experimental data is shown by filled circle while lines with different colours are used to represent models’ predictions. Solid red line is used to represent the prediction of DPMJET, blue for EPOS1.99, green for EPOS-LHC, yellow for HIJING, purple for QGSJET while dash red line is used for Sibyll.

The transverse momentum distribution of the integrated charged particles, π (π^+^ + π^−^), *K* (*K*^+^ + *K*^−^), proton (proton + anti-proton), from left to right respectively, normalized to the number of inelastic interactions are given in Fig. 1. Referring to legends in the figures, experimental data^11^ is shown by a filled circle, while lines with different colours are used to represent model predictions. Solid red line is used to represent the prediction of DPMJET-III model, blue for EPOS1.99, green for EPOS-LHC, yellow for HIJING, purple for QGSJET while dash red line is used for Sibyll. Comparing to the experimental data, DPMJET-III model predicts the yield of pions, kaons and protons for 1.2 < *p*_*T*_ < 1.7 GeV/c, 3.3 < *p*_*T*_ < 5.2, and 2.1 < *p*_*T*_ < 3.1 respectively within 10%, 15% and 15%. The model overestimates the production of pions for *p*_*T*_ < 1.2 GeV/*c* and *p*_*T*_ > 1.7 GeV/*c*, underestimates for kaons in the region other than 3.3 < *p*_*T*_ < 5.2 GeV/*c*, and for protons it predicts lower yield at *p*_*T*_ < 2.1 GeV/*c* and higher yield at *p*_*T*_. > 3.1 GeV/*c*. EPOS1.99 model describes the *p*_*T*_ distribution of the integrated yield of pion within 5%, particularly at low values of *p*_*T*_. The model reproduces the kaon and protons spectra only at low *p*_*T*_, which is within 20% for kaon and 5% for proton in 1 < *p*_*T*_ < 2 GeV/c region. The model underestimates and overestimates the spectra for kaon and proton respectively in data at high *p*_*T*_. The EPOS-LHC model predicts yield of pion within 20% at low *p*_*T*_ and within 10% at high *p*_*T*_ while predicts yields of kaon and protons only at low *p*_*T*_. EPOS-LHC model underpredicts and overpredicts respectively yield of kaon and protons at high *p*_*T*_. The HIJING model underpredicts the integrated yield of pion at low *p*_*T*_, but describes the distribution within 10% at high *p*_*T*_. The HIJING model underpredicts and overpredicts the kaons and protons yield respectively. The QGSJETII model gives higher yield of the pion as compared to the experimental data. The model predicts the kaon yield for 2.5 < *p*_*T*_ < 4.5 GeV/*c*, while describes protons yield within the uncertainty limit for 0.5 < *p*_*T*_ < 2.5 GeV/*c* but underestimates the data at high *p*_*T*_. Sibyll model overestimates (20%) the pion yield at low *p*_*T*_, but produce the results within 5% at high *p*_*T*_. The model describes Kaon distribution within 10% for *p*_*T*_ < 1.2 GeV/*c* but underestimate at high *p*_*T*_ while overestimating yield of proton over the entire *p*_*T*_ range.

**Figure 2:**
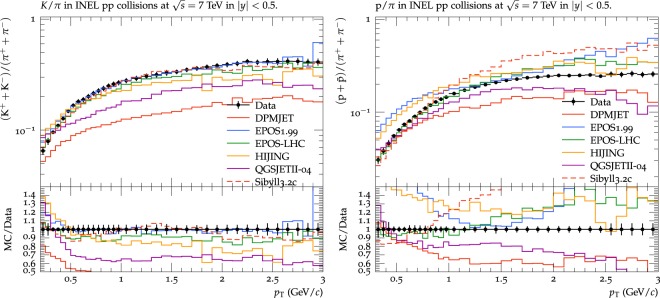
Ratio of the integrated yield of transverse momentum spectra of kaons to pions (left) and protons to pions (right) by DPMJET-III, EPOS1.99, HIJING1.383, EPOS-LHC, QGSJETII-04 and Sibyll2.3c in pp collisions at 7 TeV are compared with the measurements of experimental data from the ALICE experiment. Experimental data is shown by filled circle while lines with different colours or texture are used to represent models’ predictions as shown in legend.

The ratios of the *K* yield relative to π ((*K*^+^ + *K*^−^)/(π^+^ + π^−^)), and *p* yield relative to π ((*p* + *p*)/(π^+^ + π^−^)) are shown in Fig. 2. DPMJET-III model underpredicts both *K*/π and *p*/π ratios. EPOS1.99 model produce good prediction within 5% of the *K*/π ratio over the entire range of *p*_*T*_, but could predict only 1.2 < *p*_*T*_ < 1.7 of *p*/π ratio while overestimate the rest of the *p*_*T*_ region. EPOS-LHC model underpredicts the *K*/π ratio within 15%. The EPOS-LHC model reproduces *p*/π ratio with in 10% at *p*_*T*_ < 1.5 GeV/*c*, but overestimate at levels beyond 1.5 GeV/*c*. The HIJING model underpredicts (within 20% at low and 30% at high *p*_*T*_) the *K*/π ratio but overshoots the *p*/π ratio. QGSJETII model underpredicts both the *K*/π and *p*/π ratios. The Sibyll model predicts the *K*/π ratio very well within the uncertainty limit up to *p*_*T*_ ~ 2 GeV/*c* and within 10% at high *p*_*T*_. The model reproduces within 20% the *p*/π ratio for *p*_*T*_ < 1 GeV/*c* but overshoots the ratio afterwards.

Measurements performed in^11^ are compared with three different tunes of Pythia^41–43^, EPOS-LHC^25^ and PHOJET^44^ models. Their^11^ conclusion are summarized as follows. EPOS prediction for *p*_*T*_ spectrum of pion is reported to be within 15% with highest deviation at 1.2 GeV/c, which is more prominent for kaons and protons than pions. All three tunes of the Pythia give similar predictions for pions as given by EPOS. No one tune predicts the shape of the proton spectrum in the full *p*_*T*_ range while give a good prediction for the yield in the range 1 < *p*_*T*_ < 2 GeV/*c*, but they overshoot the measurements at lower and higher *p*_*T*_ by up to 40%. The PHOJET model fails to describes the spectrum for all the particle species. All models overshoot the kaon spectra. All models undervalue the experimental data at high momenta for the ratios *K*/π versus *p*_*T*_ with EPOS showing closest prediction. Pythia6-Z2 and Pythia8 provide good prediction of the *p*/π ratio, while all other show a large deviation at high momenta.

## Conclusion

The study compares the predictions of the transverse momentum distribution of charged particles and their ratios obtained using DPMJET-III, HIJING1.383, EPOS-LHC, QGSJETII-04 and Sibyll2.3c with the ALICE measurements obtained at mid rapidity. DPMJET-III model predicts the yield of pion, kaons and protons at mid value of *p*_*T*_ only. The EPOS1.99 and EPOS-LHC models predict the yield of pion very well while predict the yield of kaon and proton only at low *p*_*T*_. The HIJING model describes yield of pion well only at high *p*_*T*_. The QGSJETII model predicts the kaon and proton yield within the uncertainty limit of experimental data for 2.5 < *p*_*T*_ 4.5 and 0.5 < *p*_*T*_ < 2.5 respectively. Sibyll model reproduces the yield of pion at high *p*_*T*_ but overestimate the results at low *p*_*T*_. EPOS1.99 and Sibyll models with in 5% lilmit reproduce the *K*/π ratio over the entire range of *p*_*T*_ whereas the EPOS-LHC, HIJING and DPMJET model underpredict the ratio with increasing order of discrepancy respectively. The EPOS1.99, EPOS-LHC and Sibyll2.3c models predict the *p*/π ratio for low values of *p*_*T*_ whereas the DPMJET-III and HIJING models underpredict over the entire range of *p*_*T*_. Although the models’ predictions are mostly compatible with the ALICE measurements but no model completely describe all the distributions.
